# Giving a diagnosis of premature ovarian insufficiency: the importance of good communication: insights from a qualitative study

**DOI:** 10.1530/RAF-26-0076

**Published:** 2026-10-08

**Authors:** Kate Pleace, Caroline Law, Cathy Herbrand, Nicky Hudson

**Affiliations:** ^1^ Centre for Reproduction Research, De Montfort University, Leicester, United Kingdom; ^2^ Loughborough University, Loughborough, United Kingdom

**Keywords:** premature ovarian insufficiency, diagnosis, communication, qualitative research, emotional wellbeing, fertility, hormone replacement therapy, patient experience, reproductive health, counselling

## Abstract

**Objective:**

The objectives of this paper are to explore the communication of a diagnosis of premature ovarian insufficiency (POI) and to examine how the way this diagnosis is communicated affects women with the condition.

**Design:**

This is a qualitative study using semi-structured interviews and thematic analysis.

**Setting:**

Participants were recruited from across the UK and interviewed remotely using Microsoft Teams.

**Participants:**

Sixteen women aged 18 and above with a diagnosis of POI were included in this study.

**Methods:**

Participants took part in in-depth interviews exploring their experiences of living with POI, including receiving a POI diagnosis. Interviews were recorded, transcribed, and analysed thematically. This paper reports on data concerning the experience of receiving a diagnosis specifically.

**Results:**

Three key themes were identified: i) lack of information and support during the diagnosis communication, ii) emotional impact of diagnosis, and iii) framing POI as just a fertility issue. Participants reported considerable distress when diagnoses were delivered abruptly, digitally, or without appropriate support. Conversely, face-to-face, empathetic communication was described as validating and empowering. A consistent concern was the narrow clinical framing of POI as a fertility issue, with insufficient attention paid to long-term health risks, HRT, and emotional wellbeing.

**Conclusion:**

Communicating a diagnosis of POI requires a compassionate, holistic, and person-centred approach. There is an urgent need for healthcare professionals to embrace updated international guidelines, prioritise multidisciplinary care, and address existing disparities in access and support.

**Lay summary:**

Premature ovarian insufficiency (POI) occurs when the ovaries stop working normally before the age of 40. Although it is often associated with infertility, POI is also a long-term health condition that can affect bone health, heart health, menopause symptoms, emotional wellbeing, and quality of life. Receiving this diagnosis can, therefore, have a profound impact on a woman’s life. This study explored how women experienced being told they had POI. Sixteen women from across the UK took part in in-depth interviews about their experiences. Many described receiving their diagnosis in ways that felt rushed, impersonal, or lacking in compassion. Some were informed by telephone, through an online patient portal, or via printed test results without the opportunity to ask questions or receive immediate support. Women consistently reported that face-to-face consultations with clear explanations and empathy were more helpful and supportive. Participants also felt that healthcare professionals often focused mainly on fertility, with insufficient discussion about hormone replacement therapy (HRT), long-term health risks, symptom management, and emotional wellbeing. Many described feeling confused, isolated, and unprepared following their diagnosis and reported having to search for information themselves. The findings highlight the importance of delivering a diagnosis of POI with compassion, clear information, and appropriate follow-up care. Healthcare professionals should recognise that POI affects much more than fertility and ensure women receive holistic, person-centred support that addresses both the immediate emotional impact of diagnosis and the lifelong management of the condition.

## Introduction

Premature ovarian insufficiency (POI) is a condition characterised by the loss of normal ovarian function before the age of 40 (Chon *et al.* 2021), leading to reduced oestrogen production and subfertility. Unlike menopause, which typically occurs around the age of 50, POI can develop as early as the teenage years or in a woman’s twenties or thirties, presenting significant physical and emotional challenges. The condition is defined by the presence of irregular or absent menstrual cycles for at least four months, along with elevated levels of follicle-stimulating hormone (FSH) and oestrogen deficiency (Tsiligiannis *et al.* 2019). This has important implications for fertility, as ovarian function in POI may be intermittent, meaning that spontaneous conception can still occur but is often reduced, with many women who wish to have children requiring consideration of assisted reproduction options, such as egg donation (Nelson 2009).

The prevalence of POI varies across populations but is estimated to affect approximately 3% of women under the age of 40 (Li *et al.* 2023). Most cases occur spontaneously with the exact aetiology being unknown (Luisi *et al.* 2015). POI can result from genetic disorders, autoimmune diseases, infections, metabolic conditions, surgery, or medical interventions such as chemotherapy and radiation therapy (ESHRE 2024).

The health implications of POI extend beyond reproductive concerns. Life expectancy for people who have POI is two years less than those who experience menopause over the age of 55 years (Ossewaarde *et al.* 2005). Oestrogen plays a critical role in various physiological processes, and its fluctuation and deficiency can lead to a range of complications. Women with POI are at increased risk of osteoporosis due to decreased bone mineral density; in addition, oestrogen deficiency is associated with cardiovascular disease, with research suggesting that women with POI have a higher risk of heart disease compared with those who experience menopause at a typical age (Faubion *et al.* 2015). Cognitive health may also be impacted, with some studies indicating a potential link between POI and an increased risk of neurological dysfunction (Podfigurna-Stopa *et al.* 2016).

Beyond the physical effects, POI can have profound psychological and emotional consequences (Singer 2019). Many women experience grief and distress related to infertility, often accompanied by feelings of loss in relation to future family planning (Orshan *et al.* 2001). The condition is also associated with an increased prevalence of anxiety, depression, low self-esteem, and low overall life satisfaction (Maclaren & Panay 2015). The importance of timely and compassionate diagnosis has been demonstrated in a number of other areas of healthcare such as endometriosis (Denny & Mann 2008), PCOS (Bani & Seghinsara 2017), and urological conditions (Yuen-man Siu 2015) with a persistent gap reported to exist between doctors’ and patients’ understanding of patients suffering. There is limited research about how the communication of a diagnosis between the healthcare professional and patient is experienced in the context of POI (Hammond & Marczak 2025). ESHRE guidelines (2024) have highlighted the limited research on this topic and the need for further research on POI. This article presents findings from empirical research and offers a discussion on the importance of appropriate communication when giving a diagnosis of POI.

## Methods

### Study design

This study employed a qualitative design to explore women’s lived experiences of POI, including receiving a diagnosis of the condition. Qualitative approaches prioritise depth over breadth (unlike quantitative research) and focus on gaining deep insights into participants' experiences, meanings, and perspectives (Patton 2015). In-depth interviews, a common method in qualitative research, generate extensive, nuanced data that require substantial time and effort to analyse (Hoffding & Martiny 2016).

### Sample and data collection

Sixteen women aged 18 years and over with a diagnosis of POI were recruited for the study. Demographic data collected from participants were limited to age at interview, age at diagnosis, and time since diagnosis (see Table 1). Information relating to ethnicity and socioeconomic background was not collected due to Data Protection Impact Assessment (DPIA) requirements governing the scope of demographic data that could be gathered in this study.

**Table 1:** Participants’ demographic information.

Category/subcategory	Number of participants
Age (years) at interview	
18–24	3
25–34	4
35–44	5
45–54	4
Age (years) at diagnosis	
12–18	2
19–24	2
25–34	7
35–40	5
Time (years) since diagnosis	
<1	2
1–5	6
5–10	4
>10	4

Participants were recruited through online pathways, including social media platforms, POI support groups, as well as through an in-person specialist clinic, where study advertisements were posted inviting individuals to participate. Participants self-selected into the study by contacting the researcher directly. A purposive, convenience-based sampling approach was used to recruit individuals who met the inclusion criteria and were willing to share their experiences of living with POI. Inclusion criteria required participants to be aged 18 years or over, living in the UK, and to have received a diagnosis of POI. Individuals who did not meet these criteria or who were unable to participate in an interview conducted in English were excluded. Participants were offered the option of either an online or in-person interview; however, all participants chose to take part online.

A small sample size is appropriate for qualitative research as the goal is to gain depth of understanding rather than breadth, and this approach facilitates an in-depth exploration of participants’ experiences (Vasileiou *et al.* 2018). The final sample of sixteen participants was considered appropriate as it generated detailed, information-rich accounts while remaining sufficiently small to allow intensive analysis.

Semi-structured interviews were selected as this method allows both the exploration of complex topics and the opportunity to examine participants’ thoughts, feelings, and beliefs in depth (DeJonckheere & Vaughn 2019). A semi-structured interview guide was used to explore participants’ experiences, with additional probing questions asked when appropriate to encourage further depth and reflection. The ethical approval was obtained from the De Montfort University Faculty of Health and Life Sciences Ethics Committee (HLSREF 545624), and all interviews were conducted online using Microsoft Teams. Interviews lasted between 60 and 90 min and were digitally recorded using a dictaphone before being transcribed using Microsoft 365 and double-checked by the first author.

All interviews were conducted by the first author, a women’s health specialist nurse, lecturer, and researcher with both professional and her own lived experience of POI. This background provided familiarity with the condition, treatment pathways, and clinical terminology, which enhanced rapport and understanding of patient accounts, particularly pertaining to clinical and healthcare aspects. However, it was recognised that prior professional knowledge and personal experience could influence assumptions during data collection and analysis (Braun & Clarke 2022). To support reflexivity, the researcher maintained a reflective journal and recorded reflexive field notes following interviews to document emerging thoughts, assumptions, and potential biases. Reflexive awareness was maintained throughout the analytic process to ensure that interpretations remained grounded in participants’ accounts rather than shaped primarily by the researcher’s prior experiences.

Given the potentially sensitive nature of discussing experiences of diagnosis and healthcare interactions, supportive measures were implemented to minimise the risk of participant distress. Prior to the interview, participants were informed that they could pause or stop the interview at any time. During interviews, participants were reminded that they could take breaks if needed and were able to decline to answer any questions they did not feel comfortable discussing. Following the interview, participants were sent a follow-up email thanking them for their participation and providing signposting to relevant POI support resources including, support organisations, and online support groups. These measures ensured that appropriate support was available should participation raise any emotional concerns.

### Statistical analysis

From the full dataset, data pertaining specifically to being given a diagnosis of POI were extracted and then analysed using thematic analysis (King & Brooks 2018). Initial coding was conducted to identify aspects of the data of analytic interest. From these codes, themes were then developed and then reviewed, refined, and names finalised to reflect their relevance and significance in relation to the objective. This article presents data on three themes surrounding communication of a diagnosis of POI: i) lack of information and support during the diagnosis delivery, ii) emotional impact of diagnosis, and iii) framing POI as just a fertility issue.

### Results

#### Lack of information and support during the diagnosis delivery

Participants described receiving their diagnosis after investigations such as blood tests and following an initial consultation with their healthcare professional when they had first presented with symptoms such as missed periods.

Participants described receiving their diagnosis across a range of healthcare settings. Most diagnoses were delivered during consultations in primary care (*n* = 6), specialist gynaecology services (*n* = 5), or fertility clinics (*n* = 3). While most diagnoses were communicated face-to-face, three participants received their diagnosis outside of a traditional consultation, either by telephone (*n* = 1), via an online patient portal (*n* = 1), or through printed laboratory results (*n* = 1). The setting and mode of diagnosis appeared to influence participants’ experiences. Although there was no distinction in participants’ experiences according to whether the diagnosis was delivered in primary care, gynaecology, or fertility services, face-to-face consultations were consistently perceived as more supportive and conducive to understanding the diagnosis.

A frequent issue was the limited clarity and completeness of information provided at the point of diagnosis. In several cases, participants reported that the condition was not clearly named, or the key aspects, such as the clinical significance or implications for long-term health, were not adequately explained:

‘(The diagnosis) was one of the worst things that has happened to me she didn’t even call it POI.’ (P8, diagnosed in her 30s, now in her 40s)

‘I was failed, no one sat down and explained the importance of hormone replacement therapy (HRT) and possible long term health implications for POI at my diagnosis.’ (P7, diagnosed in her 30s, now in her 40s)

These accounts indicate that communication of diagnosis frequently lacked sufficient detail regarding the condition itself and its management, particularly in relation to treatment options and associated long-term health risks. This suggests a gap not only in information delivery but also in ensuring patient understanding and engagement at the point of diagnosis.

A small number of participants described receiving their diagnoses through impersonal channels such as receiving printed results or via digital health applications, without accompanying clinical support or the opportunity for discussion:

‘I was given printed results by a non-clinician with no appointment.’ (P14, diagnosed in her 30s, still in her 30s)

‘I logged into my NHS app and looked at the blood results and there was a comment saying POI, no further action, that was how I found out that I had POI.’ (P2, diagnosed in her 30s, now in her 40s)

‘The doctor told me over the phone and was so cold about it.’ (P2, diagnosed in her 30s, now in her 40s)

These examples demonstrate that diagnostic information was at times communicated without structured support, explanation, or immediate access to a healthcare professional, limiting opportunities for clarification, shared decision-making, and further follow-up. The difficulties seemed to be exacerbated when communication was not in person.

Participants frequently perceived the communication of diagnosis as overly clinical and insufficiently compassionate, highlighting the absence of emotional support during consultations.

‘When I cried, I was sent right back to the waiting room – and where do you go from there?’ (P2, diagnosed in her 30s, now in her 40s)

‘Not a positive experience, there must have been a better way?’ (P8, diagnosed in her 30s, now in her 40s)

‘The softer side wasn’t there.’ (P8, diagnosed in her 30s, now in her 40s)

Participants also highlighted a lack of coordinated follow-up care and support after the initial diagnosis. This included the absence of immediate guidance, written information, and limited signposting to resources and appropriate support networks. In some cases, individuals turned to personal networks, including friends or acquaintances, with medical expertise, to assist them with clarification and guidance.

‘Following my diagnosis a neighbour who was a GP came round on a Saturday with her textbooks and sat down with me to explain the diagnosis as I didn’t understand what I had been told by the Dr.’ (P12, diagnosed in her 20s, now in her 30s)

This quote reflects a wider tendency for participants to seek information independently, following a diagnosis, often in response to limited explanation at the point of care. This highlights a reliance on informal sources of support where formal healthcare provision was perceived as inadequate.

For the small number of participants who reported more supportive clinical interactions, these were characterised by clear explanation, time for discussion, and a proactive follow-up. Overall, the findings demonstrate a substantial variability in how POI diagnoses are delivered in clinical practice, with gaps in consistent communication, a general lack of information for newly diagnosed patients, and a lack of structured post-diagnostic support.

Participants described an unanticipated transition into a new clinical life stage for which they had not been prepared, experiencing menopausal symptoms in their teens, twenties, and thirties, a much earlier age than expected. This highlights the shortcomings in the delivery of a POI diagnosis, particularly the inadequate explanation of clinical implications and the limited provision of follow-up care.

### Emotional impact of diagnosis

Participants described a range of emotional reactions following a diagnosis of POI, most commonly characterised by feelings of shock, confusion, grief, and a profound sense of loss. For many, the diagnosis was experienced as unexpected and difficult to process, particularly when delivered with limited prior explanation or preparation. Participants frequently articulated feeling ‘broken’, ‘hopeless’, or ‘prematurely aged’:

‘I left feeling broken, confused and in shock.’ (P10, diagnosed in her 20s, now in her 30s)

‘I felt a complete failure as a woman, failure as a wife, I lost all sensuality.’ (P17, diagnosed aged 35 still in her 30s)

These quotes reflect the extent to which a diagnosis of POI was experienced not only as a medical event but as a disruption to identity, with participants describing a perceived loss of femininity, sexuality, and reproductive potential such as the loss of anticipated reproductive plans such as having their own biological children or deciding when and how to become a parent. The language used suggests that the participants often internalised the diagnosis in ways that were closely tied to social and cultural expectations of womanhood, particularly when associated with fertility, youthfulness, and normative reproductive trajectories. In this context, POI appeared to challenge deeply embedded assumptions about life course, contributing to a re-evaluation of self and future orientation.

Several participants described a sense of disconnection from their pre-diagnosis identity, consistent with experiences of biographical disruption. The diagnosis marked a clear divide on how participants understood themselves before and after their diagnosis:

‘A line in the sand of before my diagnosis and after my diagnosis, I feel like two different people.’ (P3, diagnosed in her 20s, now in her 30s)

For some, this disruption extended beyond their individual identity to affect relationships, body image, and self-esteem, particularly where diagnosis intersected with expectations around fertility and life course progression.

‘I felt that somehow I stopped being a fully functioning woman.’ (P13, diagnosed in her 30s, now in her 50s)

‘I was going to be this old woman.’ (P13, diagnosed in her 30s, now in her 50s)

Participants often described a changed relationship with their bodies, which were often seen as no longer functioning in expected or recognisable ways. This altered embodiment was often linked to feelings of disconnection, loss of control, and a sense that their bodies were not doing what they were supposed to do and aligned with their age or anticipated life trajectory. This sense of bodily disruption contributed to feelings of disconnection and uncertainty, highlighting the importance of recognising embodiment within clinical care and support.

Overall, these findings highlight the complex and multifaceted emotional impact of a POI diagnosis, encompassing both immediate reactions and long-term challenges related to identity, self-perception, and adjustment. The variability in responses also suggests that emotional outcomes may be shaped by factors such as prior symptom experience, expectations, and the context in which the diagnosis is delivered, reinforcing the need for tailored psychological and emotional support throughout diagnostic and care pathway.

### Framing POI as just a fertility issue

Many participants felt that clinical discussions during a diagnosis of POI were predominantly focused on fertility, with comparatively little attention given to the broader physical, psychological, and long-term health implications of POI.

‘There was never a conversation about treatment for symptoms, the focus was more about how I will have a family.’ (P8, diagnosed in her 30s, now in her 40s)

‘It’s not just the end of periods, it’s not just that you can’t have kids, it’s a complete transformation of your body and mind.’ (P3, diagnosed in her 20s, now in her 30s)

This emphasis on fertility often resulted in a narrowing of clinical focus, where other aspects of the condition such as symptom management, hormonal treatment, and long-term health risks were insufficiently addressed. Participants described a lack of discussion around key issues including symptoms, bone, and cardiovascular health and the ongoing management of POI as a chronic condition. Instead, fertility was positioned as the paramount consideration and the pivotal point for next steps in healthcare.

‘I was told I should run to a fertility clinic.’ (P7, diagnosed in her 20s, now in her 30s)

For some, this framing did not align with their immediate concerns or life stage, particularly where fertility was not a current priority. In these cases, the emphasis on reproductive outcomes was experienced as misaligned with individual need, limiting opportunities to address more immediate physical or psychological impacts. The prioritisation of fertility within clinical interactions also appeared to shape how participants understood their diagnosis, reinforcing a view of POI primarily in terms of reproductive loss.

‘I found out I had POI, and they offered me fertility counselling, I was 15.’ (P4, diagnosed in her teens, now in her 20s)

This narrow framing meant that some women had to do their own research to gain more of a comprehensive understanding of the condition.

‘I had to do my own research on POI, as the focus was just on having children.’ (P2, diagnosed in her 30s, now in her 40s)

Participants emphasised the importance of broader discussions around long-term health management, symptom control, and psychological support, suggesting that current approaches may insufficiently address the full scope of the patient need following a diagnosis. Taken together, these findings highlighted the need for a more comprehensive and individualised approach for communication and care, recognising POI as a complex, lifelong condition rather than solely a reproductive issue.

## Discussion

This study highlights the critical importance of the mode of communication in the diagnosis of POI and its content in terms of information regarding the condition and its possible treatments. In particular, the manner in which the diagnosis is communicated profoundly impacts patients. While there is little previous research specifically related to patients’ experiences of diagnosis of POI, research in other medical conditions such as cancer and multiple sclerosis suggests that the period of time surrounding a diagnosis can often be emotionally intense and unforgettable for the person diagnosed (Solaris 2014). Research from Solaris (2014) demonstrates that the quality of communication can either facilitate or hinder a patient’s ability to process the diagnosis, seek support, and potentially engage in long-term management of the condition.

Most participants in this study described receiving their diagnoses as impersonal, rushed, or fragmented, often delivered without explanation, context, and support, leaving them confused, overwhelmed, and unsupported. The distress this caused to women was a strong thread in their narratives regarding diagnosis. Learning about a diagnosis such as POI through a printed report, a message on a healthcare app, or a hurried telephone call challenged their sense of care or dignity. Such experiences of having a diagnosis in an insensitive manner (Singer 2012) led to confusion and feelings of abandonment. These modes of communication failed to account for the weight of what was being disclosed, not only a diagnosis but a significant and unexpected loss and a hard road to navigate (Singer 2019).

Previous research has reported an association between the time spent with clinicians when being informed of a POI diagnosis and patient satisfaction with their diagnosis (Alzubaidi *et al*. 2002). Participants in this study consistently highlighted that rushed consultations and communication failures were linked to emotional distress. By contrast, the few participants in the study who had received personalised, face-to-face explanations described these encounters as pivotal in helping them feel acknowledged and validated following their POI diagnosis. Other research from Singer (2012) and Johnston-Ataata *et al.* (2020*a,b*) emphasises the importance of positive interactions by healthcare professionals when giving a diagnosis of POI, delivering a sensitive diagnosis and giving women the opportunity to ask questions. These encounters were remembered positively, even when the news itself was distressing. Such care facilitated a stronger sense of belonging, reduced feelings of isolation, and supported engagement with future follow-up care.

The emotional responses to a diagnosis of POI often appeared to be profound. POI is a life-changing diagnosis carrying a high risk of psychosexual and psychosocial consequences (Panay *et al.* 2020). Women reported feelings of grief, devastation, shock, and significant impacts on their sense of identity, self-perception, and emotional stability. For many, POI was perceived not only as a medical condition but also as a transformative life event that redefined their sense of womanhood, ageing, and bodily autonomy, leaving some feeling ‘broken’. Previous research has indicated that a diagnosis of POI negatively affects a woman’s body image and sense of age (Groff *et al.* 2005). Unlike menopause at the expected age, which is a natural event and not a diagnosis, following a diagnosis of POI, the association of POI with infertility and premature ageing contributed to a sense of disconnection between physical experience and chronological age with many women feeling older than they were and an incongruence felt between a woman’s chronological age and her biological age (Moukhah *et al.* 2021). This psychological impact was often compounded by hormonal changes, lack of support, and inadequate information from some healthcare providers. Significant emotional and cognitive rebuilding is required due to the multiple effects of POI (Panay *et al.* 2020). These findings underscore the need for clinicians to recognise the psychological challenge and burden of a POI diagnosis and to offer appropriate emotional support at the time of diagnosis and throughout the patient’s journey.

Participants also expressed frustration that clinical conversations were often narrowly focused on fertility, a concern that has been similarly reported in other conditions such as endometriosis (Mitchell *et al.* 2024) with little attention paid to the wider implications of POI. Areas such as the importance of and need for HRT to alleviate symptoms and improve long-term health (Rahman & Panay 2021) and the significance of long-term health risks relating to cardiovascular disease, osteoporosis, and mental wellbeing (Machura *et al.* 2018) were frequently overlooked. Family building is highly individual and personal (Singer 2019), and while fertility is undeniably a significant concern for many women with POI, this singular focus failed to acknowledge the wider and lifelong consequences of the condition. This narrow clinical framing left women feeling that their concerns were not fully listened to or acknowledged and that their health needs beyond the role of reproduction were often minimised. These findings further support the need for multidisciplinary models of care that address the psychological and long-term health consequences of POI rather than just focusing predominantly on fertility.

Overall, this study demonstrates that communication is not a secondary aspect of care, but rather a vital component that can shape a woman’s ability to understand, accept, and manage a POI diagnosis (Singer 2019). For many participants, the moment of diagnosis was described not just as a medical event but as a life-altering event and turning point. The delivery of this information, whether abrupt and impersonal or compassionate and clear, had an impact on the participant.

These findings reveal dissatisfaction amongst participants with how their POI diagnosis was communicated. A lack of face-to-face consultations and insufficient explanation contributed to feelings of abandonment and confusion. It may reflect a lack of education about POI in the healthcare system. Academic medicine has acknowledged the need to teach the vital skills for communicating bad news (Rosenbaum *et al.* 2004) to assist clinicians in making a life-changing diagnosis such as POI. A diagnosis of POI has far-reaching implications across hormonal, psychological, and social domains, and gaps remain in supporting patients both at diagnosis of POI and in relation to patient education (Singleton *et al.* 2025). A more holistic and compassionate approach to communication is urgently needed, one that acknowledges the full impact of POI on a woman’s life and supports her beyond just fertility-focused care.

### Implications for clinical practice, training, and the organisation of care

The findings of this study strongly reinforce the recently updated 2024 ESHRE guidelines on POI, which advocate for early, accurate diagnosis, comprehensive patient education, and multidisciplinary care (ESHRE 2024). The guidelines offer specific information on supporting a patient with a POI diagnosis in their SPIKES protocol for guidance (Baile *et al.* 2000). These guidelines provide a structured, evidence-based approach that aligns closely with the needs of POI patients and are also voiced by the women interviewed for this study.

One of the most critical areas for improvement is the mode and manner in which the diagnosis is delivered. Clinicians must prioritise face-to-face consultations wherever possible, using language that is clear, empathetic, supportive, and providing comprehensive information about the condition and its implications and treatments. Effective communication not only improves the patient experience but also increases adherence to treatment, trust in healthcare professionals, and long-term psychological resilience.

These findings reflect broader challenges within healthcare, where reproductive conditions are often managed in siloed or specialist-focused pathways that do not reflect the complexity of women’s lived experiences. Participants’ accounts suggest that more coordinated and holistic models of care may better support women following a diagnosis of POI. Greater continuity of care and clearer follow-up pathways may also help women with POI navigate the diagnostic process and access appropriate information and support.

Participants’ accounts also reinforce the importance of multidisciplinary care following a diagnosis of POI. Although the initial diagnosis may occur in areas such as primary care, fertility services, or specialist menopause clinics, ongoing management of the condition extends beyond the initial diagnosis. While clear and comprehensive information about POI needs to be given at diagnosis, it is not realistic that all aspects and all implications of the condition can be covered in one appointment, and by one practitioner; instead, follow-up care and referrals to enable multidisciplinary care need to be put in place. Women may benefit from multidisciplinary approach to their care which may include menopause specialists, fertility specialists, counsellors, specialist nurses, and dietitians. Clear referral pathways and continuity of care are essential to ensure that women receive timely information, treatment, emotional support, and long-term follow-up throughout the life course.

The study also indicates the potential importance of further training and education for healthcare professionals involved with POI care. POI has been associated with multiple forms of losses and life disruptions for women (Davis *et al.* 2010). Yet several participants described feeling that healthcare professionals were not adequately trained or prepared to deliver news of this nature. These accounts highlight potential gaps in undergraduate and postgraduate medical, nursing, and healthcare education around the communication of complex and life-changing diagnoses such as POI. Including POI and similar conditions as part of training would not only raise awareness but also equip clinicians with the tools to respond empathetically and effectively.

### Strengths and limitations

This study offers a rich, qualitative insight into the lived experiences of women receiving a diagnosis of POI and fills a pressing gap in the literature. The online format of data collection enabled broad participation. All participants had access to digital communication, which potentially limited perspectives from those with less access or from different socioeconomic backgrounds. A strength of this study is that the length of time between being diagnosed with POI and being interviewed varied amongst participants. This ranged from recent diagnosis less than 6 months prior to interview to those with longer timelines (over 10 years between diagnosis and interview). This range allowed for the exploration of a diversity of experiences and included variance in the extent to which participants were able to reflect on their experiences of diagnosis from different time points. See Table 1.

As recruitment primarily occurred through online communities, the sample may over-represent individuals who are digitally engaged, connected to support networks and potentially health-literate (Cheng *et al*. 2022). Consequently, the experiences captured may reflect those of women who actively seek information or peer support online and may not fully represent individuals who are less connected to digital resources or support communities. These study recruitment pathways should, therefore, be considered when interpreting the findings and their transferability to the wider population of women living with POI.

The absence of broader demographic characteristics limits the ability to examine how experiences of POI and interactions with healthcare professionals may vary across different ethnic or socioeconomic groups. Previous research has highlighted that healthcare communication, access to specialist services, and diagnostic pathways can be shaped by structural inequalities, including cultural, linguistic, and socioeconomic factors (Egede *et al.* 2024). As such, the findings presented here should be interpreted with caution, as they cannot provide insights into how experiences vary across both ethnicity and socioeconomic status, and they may not capture the full diversity of experience amongst women with POI.

## Conclusion

In conclusion, this study highlights the important of good communication which includes compassion and clear information about implications, treatment, and next steps for women receiving a diagnosis of POI. Participants’ accounts suggest that gaps remain in the way this diagnosis is communicated and understood within healthcare settings. For many women receiving a diagnosis of POI represents a significant and emotionally complex moment in their journey. The way that the information is conveyed can influence whether individuals feel supported, informed, or isolated during this process.

By centring women’s experiences, this study contributes to a growing body of evidence highlighting the importance of sensitive and informed communication in reproductive healthcare. Given the qualitative and exploratory nature of the study, these findings should be interpreted as important insights into areas that may benefit from further research and professional education.

## Declaration of interest

K Pleace has previously been paid as an educational speaker for Besins UK. C Herbrand is an Associate Editor of *Reproduction & Fertility* was not involved in the review or editorial process for this paper, on which she is listed as an author.

## Funding

This research did not receive any specific grant from any funding agency in the public, commercial, or not-for-profit sector.

## Author contribution statement

KP led all aspects and was assisted with conception, planning, analysing, and writing up by CL, CH, and NH.

## Ethical approval

The ethical approval was obtained from the De Montfort University Faculty of Health and Life Sciences Ethics Committee HLSREF 545624.

## References

[bib1] Alzubaidi H, Chapin HL, Vanderhoof VH, et al. 2002 Meeting the needs of young women with secondary amenorrhea and spontaneous premature ovarian failure. J Obstectrics Gynaecol 99 P720–P725. (10.1097/00006250-200205000-00009) 11978278

[bib2] Baile W, Buckman R, Lenzi R, et al. 2000 SPIKES a six-step protocol for delivering bad news: application to the patient with cancer. Oncologist 5 P302–P311. (10.1634/theoncologist.5-4-302) 10964998

[bib3] Bani M & Seghinsara A 2017 Polycystic ovarian syndrome (PCOS) diagnostic criteria, and AMH. Asian Pac J Cancer Prev APJCP 18 P17–P21. (10.22034/APJCP.2017.18.1.17) PMC5563096 28240001

[bib4] Braun V & Clarke V 2022 Thematic Analysis: A Practical Guide. London: SAGE Publications.

[bib5] Cheng C, Gearon E, Hawkins M, et al. 2022 Digital health literacy as a predictor of awareness, engagement, and use of a national web-based personal health record: population-based survey study. J Med Internet Res 24 e35772. (10.2196/35772) PMC9526109 36112404

[bib6] Chon S, Umair Z & Mee-Sup Y 2021 Premature ovarian insufficiency: past, present and future. Mol Cell Reprod 9 672890. (10.3389/fcell.2021.672890) PMC8141617 34041247

[bib7] Davis M, Ventura J, Wieners M, et al. 2010 The psychosocial transition associated with spontaneous 46,XX primary ovarian insufficiency: illness uncertainty, stigma, goal flexibility and purpose in life as factors in emotional health. Fertil Steril 93 P2321–P2329. (10.1016/j.fertnstert.2008.12.122) PMC3013503 19243752

[bib8] DeJonckheere M & Vaughn L 2019 Semi-structured interviewing in primary care research: a balance of relationship and rigour. Fam Med Community Health 7 e000057–e000058. (10.1136/fmch-2018-000057) PMC6910737 32148704

[bib9] Denny E & Mann C 2008 Endometriosis and the primary care consultation. Eur J Obstet Gynecol Reprod Biol 139 P111–P115. (10.1016/j.ejogrb.2007.10.006) 18068292

[bib10] Egede L, Walker R & Williams J 2024 Addressing structural inequalities, structural racism, and social determinants of health: a vision for the future. J Gen Intern Med 39 487–491. (10.1007/s11606-023-08426-7) PMC10897090 37740168

[bib11] European Society of Human Reproduction and Embryology 2024 Premature Ovarian Insufficiency Guidelines. ESHRE. (https://www.eshre.eu/Guidelines-and-Legal/Guidelines/Premature-ovarian-insufficiency)

[bib12] Faubion S, Kuhle C, Shuster T, et al. 2015 Long-term health consequences of premature of early menopause and considerations for management. Climacteric 18 P483–P491. (10.3109/13697137.2015.1020484) PMC4581591 25845383

[bib13] Groff A, Covington S, Halverson L, et al. 2005 Assessing the emotional needs of women with spontaneous premature ovarian failure. J Fertil Sterility 83 P1734–P1741. (10.1016/j.fertnstert.2004.11.067) 15950644

[bib14] Hammond J & Marczak M 2025 Women’s experiences of premature ovarian insufficiency: a thematic synthesis. Psychol Health 40 P192–P216. (10.1080/08870446.2023.2192738) 36971566

[bib15] Hoffding S & Martiny K 2016 Framing a phenomenological interview: what, why and how. Phenom Congn Sci 15 P539–P564. (10.1007/s11097-015-9433-z)

[bib16] Johnston-Ataata K, Flore J & Kokanovic R 2020a Women’s experiences and treatment of early menopause and premature ovarian insufficiency: a qualitative study. Semin Reprod Med 38 P247–P255. (10.1055/s-0040-1721463) 33472244

[bib17] Johnston-Ataata K, Flore J, Kokanovic R, et al. 2020b My relationships have changed because I’ve changed’: biographical disruption, personal relationships and the formation of an early menopausal subjectivity. Sociol Health Illness 42 1516–1531. (10.1111/1467-9566.13143) 32584443

[bib18] King N & Brooks J 2018 Thematic analysis in organisational research. In The SAGE Handbook of Qualitative Business and Management Research Methods, pp 219–236. Eds C Cassell, AL Cunliffe & G Grandy. SAGE.

[bib19] Li M, Zhu Y, Wei J, et al. 2023 The global prevalence of premature ovarian insufficiency; a systematic review and meta analysis. Climacteric 26 P95–P102. (10.1080/13697137.2022.2153033) 36519275

[bib20] Luisi S, Olandini C, Regini C, et al. 2015 Premature ovarian insufficiency: from pathogenesis to clinical management. J Endocrinol Investig 1 P597–P603. (10.1007/s40618-014-0231-1) 25596661

[bib21] Machura P, Grymowicz M, Rudnicka E, et al. 2018 Premature ovarian insufficiency – hormone replacement therapy and management of long-term consequences. Menopausal Rev 17 P135–P138. (10.5114/pm.2018.78559) PMC6196774 30357030

[bib22] Maclaren K & Panay N. 2015 Current concepts in premature ovarian insufficiency. Women’s Health 11 P169–P182. (10.2217/whe.14.82) 25776291

[bib23] Mitchell AM, Rajapakse D, Peate M, et al. 2024 The ‘most bothersome symptom’ construct: a qualitative study of Australians living with endometriosis. Acta Obstet Gynecol Scandinavia 103 P1625–P1633. (10.1111/aogs.14876) PMC11266636 38751074

[bib24] Moukhah S, Ghourbani B, Behboodi-Moghadam Z, et al. 2021 Perceptions and experiences of women with premature ovarian insufficiency about sexual health and reproductive health. BMC Womens Health 21 54. (10.1186/s12905-021-01197-5) PMC7869211 33557799

[bib25] Nelson L 2009 Primary ovarian insufficiency. N Engl J Med 360 P606–P614. (10.1056/nejmcp0808697) PMC2762081 19196677

[bib26] Orshan A, Furness K & Frost C 2001 The lived experience of premature ovarian failure. J Obstet Gynecol 30 P202–P208. (10.1111/j.1552-6909.2001.tb01536.x) 11308110

[bib27] Ossewaarde M, Bots M, Verbeek A, et al. 2005 Age at menopause, cause-specific mortality and total life expectancy. J Epidemiol 16 P556–P562. (10.1097/01.ede.0000165392.35273.d4) 15951675

[bib28] Panay N, Anderson RA, Nappi RE, et al. 2020 Premature ovarian insufficiency: an International Menopause Society White Paper. Climacteric 23 P426–P446. (10.1080/13697137.2020.1804547) 32896176

[bib29] Patton M 2015 Qualitative Research and Evaluation Methods: Integrating Theory and Practice, 4th edn. Thousand Oak, California: Sage Publications.

[bib30] Podfigurna-Stopa A, Czyzyk A, Grymowicz M, et al. 2016 Premature ovarian insufficiency: the context of long-term effects. J Endocrinol Investig 39 P983–P990. (10.1007/s40618-016-0467-z) PMC4987394 27091671

[bib31] Rahman R & Panay N 2021 Diagnoses and management of premature ovarian insufficiency. Best Pract Res Clin Endocrinol Metabilism 35 101600. (10.1016/j.beem.2021.101600) 34823999

[bib32] Rosenbaum M, Ferguson J & Lobas J 2004 Teaching medical students and residents skills delivering bad news: a review of strategies. Acad Med 79 P107–P117. (10.1097/00001888-200402000-00002) 14744709

[bib33] Singer D 2019 Managing the psychological sequelae of POI. Post Reprod Health 25 P150–P155. (10.1177/2053369119874640) 31630612

[bib34] Singer D 2012 ‘Its not supposed to be this way’; psychological aspects of premature menopause. Counsell Psychother Res J 12 P100–P108. (10.1080/14733145.2011.648202)

[bib35] Singleton A, Hipp H, Ali N, et al. 2025 Women’s healthcare providers’ knowledge and practices surrounding fragile-X associated premature ovarian insufficiency (FXPOI). J Assist Reprod Genet 42 P499–P508. (10.1007/s10815-024-03337-w) PMC11871246 39641836

[bib36] Siu JY. 2015 Communicating under medical patriarchy: gendered doctor-patient communication between female patients with overactive bladder and male urologists in Hong Kong. BMC Womens Health 15 44. (10.1186/s12905-015-0203-4) PMC4448299 26021313

[bib37] Solari A 2014 Effective communication at the point of multiple sclerosis diagnosis. Mult Scler J 20 P397–P402. (10.1177/1352458514523061) 24503056

[bib38] Tsiligiannis S, Panay N & Stevenson J 2019 Premature ovarian insufficiency and long-term health consequences. Curr Vasc Pharmacol 17 P604–P609. (10.2174/1570161117666190122101611) 30819073

[bib39] Vasileiou K, Barnett J, Thorpe S, et al. 2018 Characterising and justifying sample size sufficiency in interview-based studies: systematic analysis of qualitative health research over a 15-year period. BMC Med Res Methodol 18 148. (10.1186/s12874-018-0594-7) PMC6249736 30463515

